# Supplementary material for: Nucleation-dependent propagation of Polycomb modifications emerges during the Drosophila maternal to zygotic transition
Source: eLife. 2026 Aug 13;14:RP108371. doi: 10.7554/eLife.108371 (PMC13472630; doi:10.7554/eLife.108371)
Supplement: Peer review history

# Editor report: eLife Assessment

**DOI:** 10.7554/eLife.108371.3.sa0

**Authors:** Xin Chen

**Affiliations:** Johns Hopkins University United States

**Keywords:** Compelling, Fundamental

This study employs state-of-the-art quantitative imaging and genomics approaches to address a **fundamental** question regarding the establishment of Polycomb domains during Drosophila embryogenesis. The authors identify the maternal-to-zygotic transition, rather than earlier developmental stages, as the critical window for Polycomb domain establishment, providing significant clarification of the timing of this process and they further investigate the roles of Zelda and GAGA factor, demonstrating that Zelda, but not GAGA factor, contributes to Polycomb domain establishment. Overall, the conclusions are well supported by the experimental evidence presented. These **compelling** findings advance our understanding of how Polycomb domains are established during early embryogenesis and have broad implications for chromatin regulation and developmental biology.

# Referee report: Reviewer #3 (Public review)

**DOI:** 10.7554/eLife.108371.3.sa1

Gonzaga-Saavedra et al report an analysis on genomic binding of Polycomb group proteins, and of H2Aub1 and H3K27me3 domain formation in the early Drosophila embryo. Using carefully stage embryos during the nuclear cycles (NC) leading up to the cellular blastoderm stage, the authors provide compelling evidence that H3K27me3 domains at PcG target genes are only established during NC14 and do not exist in NC13. In contrast, H2Aub1 domains already start to appear during NC13. The authors show that E(z), the catalytic subunit of the H3K27 histone methyltransferase PRC2, is readily detected in interphase nuclei during the rapid nuclear divisions in pre-blastoderm embryos. In contrast, the DNA-binding proteins Pho, Cg and GAF that are known (Pho) or have been postulated (Cg, GAF) to anchor PRC2 and PRC1 to Polycomb Response Elements (PREs) in Polycomb target genes only start to show nuclear localization from NC10 onwards with gradually increasing nuclear concentrations, reaching a maximum during NC14. These data strongly corroborate the simple straightforward view that targeting of PRC2 and PRC1 to PREs by sequence-specific DNA-binding proteins is a pre-requisite for the formation of H3K27me3 and H2Aub1 domains at Polycomb target genes.

The authors then explore the potential role of GAF/Trl in this process. They find that in embryos depleted of GAF/Trl, H3K27me3 domain formation is largely unperturbed.

The authors also depleted the pioneer factor Zelda (Zld) and found that removal of Zld results in a more complex outcome. Zelda appears to counteract accumulation of H3K27me3 at the Polycomb targets eve and zen but also appears to be required for effective H3K27me3 domain formation at Polycomb targets such as amos or atonal.

This is a very thorough study that reports data of superior technical quality that are highly relevant for the field. The study by Gonzaga-Saavedra et al extends and strengthens previous work from the labs of Eisen (Li et al, eLife 2014) and Zeitlinger (Chen et al, eLife 2013) to convincingly demonstrate that Polycomb domain formation in the early embryo occurs during ZGA but that such domains do not exist prior to ZGA. This should now finally put to rest earlier claims by the Iovino lab (Zenk et al, Science 2017) that H3K27me3 domains present in the zygote nucleus would be propagated and partially maintained during the rapid nuclear cleavage cycles and serve as seeds for H3K27me3 domain formation during ZGA.

The experiments analyzing H3K27me3 domain formation in embryos depleted of GAF/Trl or Zelda will be of great interest to the field.

Comments on revised version.

In the revised version, the authors have addressed the comments and suggestions raised by this reviewer and added the missing references to earlier work.

# Author comment: Author response

**DOI:** 10.7554/eLife.108371.3.sa2

**Authors:** Natalie Gonzaga-Saavedra, Eleanor A Degen, Isabella V Soluri, Corinne Croslyn, Shelby A Blythe

**Affiliations:** Department of Molecular Biosciences, Northwestern University Evanston United States; Department of Molecular Biosciences, Northwestern University Evanston United States; Northwestern University Evanston United States; Department of Molecular Biosciences, Northwestern University Evanston United States; Department of Molecular Biosciences, Northwestern University Evanston United States

The following is the authors’ response to the original reviews.

**Public Reviews:**

**Reviewer #1 (Public review):**

This well-conceived manuscript investigates the mechanisms that shape the chromatin landscape following fertilization, using the Drosophila embryo as a model system. Importantly, the authors revisit conflicting data using new approaches and analysis to show that the silent H3K27me3 mark deposited by PRC2 is established de novo in the embryo in coordination with the slowing of the nuclear division cycle and activation of zygotic transcription. Unexpectedly, they demonstrate that the transcription factor GAF is not required for the deposition of this mark, but that the well-studied pioneer factor Zelda, which is required for widespread gene expression, is required for H3K27me3 deposition at a subset of regions. The experiments are rigorously performed, and interpretations are clear. Strengths of this manuscript include the rigor of the experimental design, careful analysis, and well-supported conclusions. Some additional citations, analysis, and broadening of the Discussion section to include additional models and data would further strengthen this manuscript.

We appreciate the reviewer’s positive assessment and in revision we have revised the Discussion to clarify some of the mechanistic insights of the work as well as including references to related studies in the zebrafish model system.

**Reviewer #2 (Public review):**

Summary:

Epigenetic silencing of target genes by the Polycomb pathway is central to maintenance of cell fates during development and depends on repressive chromatin states involving Polycomb complexes and histone modifications. However, the mechanisms by which these chromatin states are built at the earliest stages of development are unclear. Here, Gonzaga-Saavedra and colleagues use the premier experimental system for studying Polycomb gene regulation, Drosophila development, to investigate when Polycomb domains emerge and how they are assembled. Using a combination of CRISPR gene editing, imaging, and genomic profiling, they determine that while H3K27me3 is initially present in the first nuclear cycles, it quickly dissipates and does not re-emerge until mid-nuclear cycle 14, during the major wave of zygotic genome activation (ZGA). This finding helps resolve current discrepancies in the field, informs potential mechanisms of transgenerational inheritance, and indicates that repressive Polycomb domains are built de novo on target genes in embryogenesis. The authors then set out to examine how Polycomb domains are built. Through live imaging and immunofluorescence, they determine that the histone H3K27 methyltransferase, E(z), is present in nuclei at high levels throughout cleavage and blastoderm stages. By contrast, they determine that several Polycomb proteins that bind PREs (cis elements that demarcate Polycomb targets in the genome) are absent from early cleavage nuclei and progressively increase following nuclear cycle 10. These findings suggest that the absence of H3K27me3 in early embryos may be due to failure to assemble functional Polycomb complexes at target genes. Lastly, the authors test the requirement of two transcription factors with important roles in ZGA, GAF, and ZLD. Despite binding to many PREs and regulating chromatin accessibility in early embryos, they find that GAF is largely dispensable for the emergence of H3K27me3 domains. On the other hand, they find that the pioneer factor ZLD is required for proper H3K27me3 emergence; in its absence, some Polycomb domains accumulate greater levels of H3K27me3, whereas other Polycomb domains accumulate less H3K27me3.

Strengths:

The strengths of this study are manifold. It studies an important topic with broad interest to the chromatin and epigenetics fields. It is well-written with detailed method descriptions. In addition, the experimental design and rigor of execution are exceptional despite working with very small amounts of biological material. Example strengths include that the Polycomb proteins studied were tagged with the same epitope, permitting direct quantitative comparisons in imaging and in genomics experiments. Microscopy studies are quantified and performed both via live imaging and via immunofluorescence. The microscopy studies reinforce and extend conclusions made via ChIP. Sophisticated loss-of-function analyses allow for direct mechanistic tests of Polycomb domain emergence.

Weaknesses:

Overall, the study is quite strong already, but it can be further strengthened in several ways. First, several conclusions should be refined based on the data presented. Second, the extent to which ZLD is important for initiating Polycomb domain formation should be made clearer. Third, additional genomic profiling experiments are needed to provide insight into models explaining why H3K27me3 is absent prior to NC14.

We are grateful for the reviewer’s thorough and supportive comments. We have revised certain assertions and conclusions for objectivity. For the point about providing “insight into models explaining why H3K27me3 is absent prior to NC14,” we have a separate study that addresses this issue directly (Degen, Gonzaga-Saavedra, and Blythe, bioRxiv 2025, in press). In summary, we find evidence that a maternal PcG imprint is indeed maintained through cleavage divisions, albeit through lower-order methylation states (maximally, H3K27me2). We chose not to include these additional results in this manuscript to maintain the focus of this study on ZGA. Our revision of the manuscript includes a reference to this associated study in the Discussion.

**Reviewer #3 (Public review):**

Gonzaga-Saavedra et al report an analysis on genomic binding of Polycomb group proteins, and of H2Aub1 and H3K27me3 domain formation in the early Drosophila embryo. Using carefully staged embryos during the nuclear cycles (NC) leading up to the cellular blastoderm stage, the authors provide compelling evidence that H3K27me3 domains at PcG target genes are only established during NC14 and do not exist in NC13. In contrast, H2Aub1 domains already start to appear during NC13. The authors show that E(z), the catalytic subunit of the H3K27 histone methyltransferase PRC2, is readily detected in interphase nuclei during the rapid nuclear divisions in pre-blastoderm embryos. In contrast, the DNA-binding proteins Pho, Cg, and GAF that are known (Pho) or have been postulated (Cg, GAF) to anchor PRC2 and PRC1 to Polycomb Response Elements (PREs) in Polycomb target genes only start to show nuclear localization from NC10 onwards with gradually increasing nuclear concentrations, reaching a maximum during NC14. These data strongly corroborate the simple, straightforward view that targeting of PRC2 and PRC1 to PREs by sequence-specific DNA-binding proteins is a prerequisite for the formation of H3K27me3 and H2Aub1 domains at Polycomb target genes.

The authors then explore the potential role of GAF/Trl in this process. They find that in embryos depleted of GAF/Trl, H3K27me3 domain formation is largely unperturbed.

The authors also depleted the pioneer factor Zelda (Zld) and found that removal of Zld results in a more complex outcome. Zelda appears to counteract the accumulation of H3K27me3 at the Polycomb targets eve and zen, but also appears to be required for effective H3K27me3 domain formation at Polycomb targets such as amos or atonal.

This is a very thorough study that reports data of superior technical quality that are highly relevant for the field. The study by Gonzaga-Saavedra et al extends and strengthens previous work from the labs of Eisen (Li et al, eLife 2014) and Zeitlinger (Chen et al, eLife 2013) to convincingly demonstrate that Polycomb domain formation in the early embryo occurs during ZGA but that such domains do not exist prior to ZGA. This should now finally put to rest earlier claims by the Iovino lab (Zenk et al, Science 2017) that H3K27me3 domains present in the zygote nucleus would be propagated and partially maintained during the rapid nuclear cleavage cycles and serve as seeds for H3K27me3 domain formation during ZGA.

The experiments analyzing H3K27me3 domain formation in embryos depleted of GAF/Trl or Zelda will be of great interest to the field.

We thank the reviewer for recognizing the strength of our data and conclusions, and we agree that our results help settle conflicting claims in the field. We have emphasized Zelda’s context-dependent effects more clearly in the revised manuscript.

**Recommendations for the authors:**

**Reviewing Editor Comments:**

It would strengthen the manuscript to more fully acknowledge and discuss related work in the field. Addressing the caveats in the functional analyses, either through editorial clarification or additional experiments, would also improve the study.

For recommendations to the authors, comments from each reviewer are listed below.

**Reviewer #1 (Recommendations for the authors):**

Some additional analysis would clarify the relationship between pPREs and transcription factors.

Because of the limitations of depleting Pho, the model that nuclear levels of Pho, Cg, and GAF regulated E(z) activity is purely correlative, as GAF depletion did not change H3K27me3 distribution. As it stands, it is possible that this NC14 nuclear enrichment of these factors is not relevant. The statement on lines 295-297 regarding the correlation between re-establishment of the modification state and nuclear localization of Pho, Cg, and GAF is true, but a bit misleading since there is no evidence to support the necessity of these factors for H3K27me3 establishment. Other models remain possible, and the discussion should be toned down to account for this. Furthermore, the only factor that is shown to influence H3K27me3 is Zelda, which does not show an increase in nuclear localization at NC14.

We thank the reviewer for highlighting this issue. As the reviewer indicates, we lack definitive mechanistic evidence that limited nuclear localization of any recruitment/nucleating factor is limiting for H3K27me3 deposition. However, our data are, we feel, definitive in terms of demonstrating that nucleation from PREs and PRE-like regions arises at mid-NC14 for H3K27me3, and in late cleavages for H2Aub. Ideally, we would test each known nucleating factor for a necessary role in mediating this activity. We have chosen not to include such measurements in this manuscript because a proper mechanistic treatment of each factor (Pho, Cg, and others) would need to be extensive, and we feel better suited for an independent study. We have added language to the “Limitations of the Study” section to reflect the remaining need to identify the key nucleating factors responsible for establishment of the zygotic PcG landscape.

We re-read the lines the reviewer suggested were misleading and we respectfully disagree. The lines (“Taken together, these observations are consistent with a model where…re-establishment of this modification state is restricted to late cleavage divisions by limiting nuclear localization of nucleating factors such as Pho, Cg, and GAF.”) are expressing a hypothesis/model, and in our opinion these lines are suitably framed as to not be misleading.

Additional analysis/discussion regarding the relationship between Zelda, H3K27me3, CBP, and paused polymerase would provide further clarity into how Zelda might promote this methylation. It would be useful to discuss how the various classes defined correlate with enhancers versus promoters, and also the gene expression of the underlying gene. It would be clarifying to discuss how the H3K27me3 at these Zelda-dependent regions relates to gene silencing since many of the genes depend on Zelda for expression. Are these genes expressed prior to NC14 and then silenced at this time point? How do H3K27ac levels, which Zelda promotes through recruitment of CBP, relate to the various pPRE classes? The authors should also consider the report from the Mannervik lab that CBP is instrumental in promoting H3K27me3 (Hunt, Boija, Mannervik et al. Mol Cell 2022 82:3580-3597) and the relationship with paused RNA Pol II. Given this possible connection, it could be useful to consider that paused polymerase is also first evident at NC13/14. Overlaying the pPREs with paused polymerase from Chen et al. 2013 eLife (current citation 30) could be informative. At a minimum, a discussion of these additional mechanisms and the implications of the data in Hunt et al. would strengthen the Discussion section.

We thank the reviewer for this comment. We agree that additional analysis of the relationship between Zelda, H3K27me3 and CBP would be essential for providing mechanistic clarity on the role of Zelda for putting these loci into play for apparently either positive or negative regulation. At present, we feel that extensive additional analysis, including work with CBP, PRC1, and nucleating factors such as Pho would be better suited for a future study.

H2Aub is clear earlier in development than H3K27me3, and in mice and zebrafish, it promotes PRC2-mediated H3K27me3 (Hickey et al. eLife 2022 doi: 10.7554/eLife.67738, citations 86, 89). As such, it remains possible that this mark is instructive for the H3K27me3 deposition observed. As such, a bit more analysis of where H2Aub is deposited and how it overlaps with pPREs might help determine whether similar mechanisms could be important in Drosophila.

We suspect that similar mechanisms are important in Drosophila as well. The omission of the Hickey…Cairns reference was an oversight in the original document. We have revised this sentence to refer to both mouse and zebrafish and have added the citation.

Prior work has noted the sudden increase in GAF nuclear concentration. Please cite Dima and Reeves. Development 2025 152:dev204460 in support of the observations shown in Figure 3D.

Thank you. Yes, this article was published shortly after we submitted this manuscript for review and we have now added it to reflect its independent replication of the GAF nuclear concentration result.

It is not clear that Figure 4 warrants an entirely new figure, since the conclusions drawn are similar to/the same as Figure 3. Perhaps change to a supporting figure?

We agree that this figure was repetitive. We have now moved it to a figure supplement of Figure 3.

The authors have developed a powerful modified ChIP protocol that enables them to perform the experiment on the equivalent of 10 embryos! This is not highlighted in the manuscript, despite the vast improvement this provides. The authors should highlight this in the manuscript, unless a separate manuscript describing this technique is being written/published. Regardless, this is a very exciting protocol.

Thank you. A methods paper describing this approach is in preparation.

Minor:

(1) Line 49-53: clarify that, as opposed to the mechanisms described earlier in the paragraph, these mechanisms are specific to Drosophila.

Done.

(2) Line 61: cite Sun et al. and Schulz et al. (current citations 60 and 61) since these papers demonstrated the pioneering function of Zelda.

Done.

(3) Line 95: a word seems to be missing. Perhaps "approach (STAN) to identify a set of PcG "domains" from our"?

We have made this revision.

(4) Line 217: Calling an embryo a "specimen" is odd. Can you just say all embryos?

Ok.

(5) Line 316: GAF is encoded by Trithorax-like, not Trithorax-related.

Revised.

(6) Figure 5: The arrowheads and asterisk are so small that they are nearly impossible to see when the figure is printed. Please make it larger and perhaps use a color that stands out more.

We have made the arrowheads and asterisk larger and changed the color to yellow to improve visibility.

**Reviewer #2 (Recommendations for the authors):**

(1) Regarding weakness 1, the title states "nucleation-dependent propagation". This is an overstatement of the study's conclusions because these features are inferred and not directly tested here. This is an easy fix with text revisions.

We acknowledge that we have not directly tested the role of specific nucleating factors for the process of nucleation-dependent propagation. This is reflected in the Discussion text. We have added to the “Limitations of the Study” section the following text: “Finally, we acknowledge that further loss-of-function analysis will be necessary to determine the key nucleating factors responsible for the initial establishment of the zygotic H2Aub and H3K27me3 landscape.” However, we feel that we have demonstrated definitively that, by mid-NC14, nucleation of H3K27me3 sites is first detected genome-wide. As such, we have left the title as-is.

(2) Also, regarding weakness 1, the abstract makes additional overstatements. These are easy fixes with text revisions.

(a) "A large subset of targets requires ZLD..." (line 24). This phrase makes it sound like the majority of Polycomb domains depend on ZLD, which I am not sure is accurate.

Thank you for pointing this out. We agree with this assessment and have revised the abstract to remove the word “large” so that now the sentence reads “; a subset of targets requires Zelda…”

(b) "...requires ZLD not for PcG factor recruitment" (same sentence). This sentence should state "E(z)" instead of "PcG factor" because E(z) was the only one tested in ZLD mutants.

We agree as well and have made the requested change to exchange “PcG factor” to “E(z)”.

(c) "to license a loaded PRE" (same sentence). Whether PREs are fully loaded in ZLD mutants was not tested. In addition, see comments below for feedback on the use of the term "license."

We have revised this to read “to license an E(z)-loaded PRE,” to keep consistent with the above suggested change. We also removed the mention of “H2Aub” because now the PRE-loading acknowledges we have only measured E(z), although we see effects on both modifications. We acknowledge, in light of Reviewer 3’s comment, that we have not directly measured whether PRC1 still can bind to certain PREs in the absence of Zelda like we see with E(z)/PRC2.

(3) Also regarding weakness 1, the authors set up a dichotomy for ZLD's role in initiating Polycomb domain formation, either acting as a pioneer or as a licensing factor. However, based on the data presented (e.g. the atonal browser shot), it appears that chromatin accessibility is lost in both sub-classes of H3K27me3 domains that depend on ZLD, meaning that ZLD's role as a pioneer would explain both sub-classes, and that a licensing role is not supported by the data. To assess whether there are pioneer-independent roles of ZLD in the emergence of Polycomb domains, it may help to examine the role of chromatin accessibility changes directly (e.g., is there a substantial fraction of changes in H3K27me3 domains or E(z) peaks that cannot be attributed to changes in chromatin accessibility in ZLD mutants?). The authors should refine their language or provide additional support for the non-pioneering role of ZLD.

We acknowledge that the pioneer/licensing distinction is still not clear from a mechanistic perspective and that future work will be needed to address this issue. We do not mean to imply that the licensing is necessarily independent of pioneering, rather that at sites like *atonal*, clearly chromatin accessibility is not the only job that Zelda performs. There are at least two possible mechanisms: (1) it is all about pioneering, and in the absence of accessible chromatin, some other factor required for stimulating H3K27me3 deposition is unable to bind; or (2) it is all about Zelda, and in mutants Zelda both does not confer accessible chromatin, and also does not stimulate H3K27me3 deposition through whatever means. For either mechanism, the remarkable feature is that E(z) still localizes to its genomic target and requires additional information to deposit H3K27me3. This is distinct from the other class (represented by amos in Figure 6 in the final manuscript) where loss of accessibility correlates with loss of E(z) –and presumably PRC2– binding. To explain this activity, we have invoked the term “licensing” which we feel captures the effect of Zelda (whether it be direct or indirect). The possibility of indirectness is a significant caveat, so we have added clarifying text to indicate this possibility. We have added to paragraph 2 of the Discussion the sentence, “As such, we emphasize that Zelda-dependent licensing could stem either directly or indirectly from Zelda function.”

(4) Regarding weakness 2, as currently written, it seems like a small fraction of H3K27me3 domains depend on ZLD. Is this accurate? Some of my confusion may stem from alternating use of bins and runs. Can the fraction of domains that depend on ZLD be made more explicit through text revisions and additional bioinformatics? More comprehensive bioinformatics analyses can be performed with the ZLD mutant datasets by incorporating ATAC and E(z) peaks. For instance, what fraction of E(z) peaks inside and outside of Polycomb domains are affected in ZLD mutants, and do these E(z) changes correlate with H3K27me3 changes? Similarly, what fraction of PREs change in accessibility in ZLD mutants, and are these accessibility changes correlated with H3K27me3 changes? When do PREs become accessible during wild-type embryogenesis? Are there unique features of ZLD-dependent H3K27me3 domains or E(z) peaks? The authors' perspective on the extent to which ZLD is required for Polycomb domain initiation should also be added to the Discussion.

We find that 38 PcG domains are sensitive to Zelda (9 have increases, 29 have decreases in H3K27me3), and this is stated in the Results. This accounts for 16% of domains, which is a small fraction of the total. We have added a sentence to the results reporting this fraction. An earlier draft of this manuscript included an analysis of accessibility: both in terms of the timing of when PREs gain accessibility and their dependency on Zelda function. This section was omitted from the submitted manuscript because it did not add clarity the distinction between classes that we report. As for the final request that we add to the Discussion our perspective on the extent to which Zelda is required for PcG domain initiation, we now address this in the second paragraph of the Discussion.

(5) Regarding weakness 3 (why is H3K27me3 missing pre NC14?), it is suggested that the absence of H3K27me3 is due to the short duration of nuclear cycles relative to the rate of me2->me3 catalysis. And although the authors suggest that Polycomb complex assembly occurs at pPREs without H3K27me3, their microscopy studies indicate that binding of Polycomb proteins to PREs may be regulated via nuclear accumulation. Therefore, it remains unclear whether (and when) the lack of H3K27me3 is due to incomplete Polycomb complex assembly. Additional genomic profiling experiments are needed to directly test when Polycomb complexes assemble on chromatin relative to the emergence of H3K27me3 domains. These experiments would also support claims of nucleation.

(a) ChIP of E(z) and a PRE binding protein (Pho, Gc, GAF) should be performed at NC13 to test whether Polycomb proteins (especially PRC2) are bound at PREs prior to H3K27me3 emergence.

(b) ChIP of E(z) should also be performed at NC10 when the PRE binding proteins appear to be absent but when E(z) is hyperabundant in the nucleus. Can E(z) bind PREs in the absence of these "nucleators"? Or does E(z) promiscuously interact with chromatin, helping to explain the broad, low-level H3K27me1 enrichment profile?

We thank the reviewer for this comment. We have addressed this issue in a separate study (preprinted and accepted for publication as of this writing). Although H3K27me3 is not detectable on cleavage-stage chromatin, lower-order H3K27me2 is maintained on chromatin and detected throughout cleavages by immunostaining and by ChIP. The maintenance of cleavage-stage H3K27me2 depends on both E(z) and Esc. Notably, this lower-order state reflects maintenance of a maternally supplied H3K27 methylation state: H3K27me2 is only detected on maternal (not paternal) chromatin during the period (prior to NC10) when Pho/Cg/GAF do not localize to nuclei. Overall, these observations underscore the limiting nature of early cleavages to support de novo establishment of H3K27 methyl states, but also demonstrate the competency of the PcG system during this time to engage in some degree of H3K27 maintenance.

Minor Comments:

(1) It is interesting that the great majority of E(z) peaks (72%) are outside of H3K27me3 domains at NC14. What are these sites? Do these sites correspond to Polycomb domains later in embryogenesis (ie, do they become marked by H3K27me3 later)? Or, do these E(z) peaks disappear at later stages of embryogenesis?

We agree that this observation is interesting. Figure 2 shows that the majority of these sites are concurrent with transcription start sites. Visual comparison between ChIP datasets generated here and any of the various publicly available datasets generated at later stages (e.g., stage 16 embryos) reveals that the set of PcG domains observed at ZGA is fairly consistent with the set of PcG domains observed later on. Therefore, on a bulk level, these extra-domain E(z) sites observed at ZGA do not represent later-onset PcG domains. However, at this level of resolution, we cannot rule out that in some cell type these sites are converted to a cell-type-specific PcG domain. We have not addressed the perdurance of these E(z) peaks at later stages. The function and the fate of these extra-domain binding sites remains an open question for future investigation.

(2) Figure 3A live imaging indicates that E(z) nuclear signal intensity diminishes over subsequent nuclear cycles. Can the authors expand on whether photobleaching may contribute to this decrease? The E(z) signal in IF experiments should be quantified to test whether it also diminishes over successive nuclear cycles.

The imaging conditions for this experiment were controlled to minimize photobleaching in the EGFP channel. While we cannot rule out some small contribution of photobleaching to the overall signal intensity, the overall trend reported in the quantification of Figure 3A reflects, to the best of our abilities to measure, a biological effect. This effect is also evident in the immunofluorescence imaging without need for additional quantification: From NC10 to NC14, when nuclei are presented on the embryo surface, a clear decrease in staining intensity is observed (Figure 3-figure supplement 1A in the final manuscript). Our observations indicate that E(z) decreases in concentration with increasing nuclear content during the cleavage divisions.

(3) Can the authors provide further interpretation of the H3K27me1 signal profile? There is very little difference in signal amplitude inside relative to outside domains in Figure 1C, and across a 20kb window surrounding E(z) peaks in Figure 1B. Is all this chromatin considered to be H3K27me1-enriched, or is there a high level of noise? The anticorrelation with H3K27me3 at NC14 is compelling and seems to argue that the broad H3K27me1 signal is real.

The H3K27me1 signal profile reflects a likely broad distribution of H3K27me1 that includes not only canonical “PcG domains” (i.e., regions that ultimately gain high-level H3K27me3) but also non-canonical domains. Consistent with observations in other systems (e.g., PMID: 24289921), H3K27me1 is broadly distributed across the Drosophila genome. We have added the following sentence to the Results section to contextualize our description of the H3K27me1 domains: “The broad genome-wide distribution of H3K27me1, including in regions outside of canonical PcG domains, resembles profiles previously measured in mammalian tissue culture.” (citing the Ferrari et al study).

**Reviewer #3 (Recommendations for the authors):**

My suggestions for changes are mainly of an editorial nature.

My comments below are not in order of priority, but grouped into different types of suggestions for changes.

Comments on the presentation of results:

(1) The relationship between the genomic regions shown in the heat map in Figure 1A and Figure 2A is not clear. In Figure 2A, 1264 regions with E(z) peaks in PcG/H3K27me3 domains are shown. In Figure 1A, the text (line 97/98) states that 237 PcG/H3K27me3 domains contain 1 or more E(z) peaks, and on the left of the H3K27me3 heat map panel, it says "PcG domains". Are these then the 237 regions that are shown in Figure 1A? Only the top ones seem to have high levels of H3K27me3. Please clarify.

We could have been more clear about this. As the reviewer points out, there are different numbers of peaks shown in the heatmaps in Figures 1A and 2A. Figures 1A and B plot one E(z) peak per domain, determined by finding the one maximal E(z) peak per and plotting it. This is stated in the figure legend: “One representative E(z) peak per domain was selected for plotting.” Figure 2A plots all E(z) peaks within Domains (n = 1264). For the heatmaps and the average plots in Figures 1A and 1B, we found that selecting the maximal E(z) peak yielded a more accurate representation of the accumulation of H3K27me1/3, compared with plotting this for all E(z) peaks within domains, presumably because some called peaks (e.g., many of the minor E(z) peaks shown in Figure 1E) do not appear to be the primary sites of nucleation within the domain. For Figure 2, we wished to compare all E(z) peaks, inside and outside of domains, and for this we plotted heatmaps over all of the peaks.

(2) In general, in the text, the figure panels should be discussed in order of appearance. For example, it is not ideal that after describing the data in Figure 1A, the text jumps to discuss results shown in Figure 2A and only then goes back to discuss Figure 1B and C. This should be easy to resolve by reorganizing the text or perhaps re-arranging figure panels (e.g., perhaps moving elements to additional supplemental figures?).

We acknowledge that this is inconvenient and we apologize for this. However, we feel the flow of the manuscript and the figures themselves benefit from this somewhat awkward order of discussion and we have chosen to leave them as-is.

(3) In general, I felt that the text could be improved by putting some of the more detailed technical procedures and result descriptions into Materials and Methods or Figure legends in order not to disrupt the flow of the text.

Comments for improving discussion:

(4) When citing and discussing the previous literature that claimed a function for GAGA factor (GAF/ Trl) in Polycomb repression (i.e., refs. 12, 14, 49-52) on page 15/16 and again on page 25, the authors may also want to cite earlier studies that failed to observe a function of GAF/Trl in Polycomb repression. Specifically, previous studies (Brown et al, Development 2003) had investigated a possible role of GAF/Trl in Polycomb repression in larvae using stringent tests (analysis of HOX gene expression in Trl null mutant cell clones in imaginal discs, removing Trl in a sensitized pho null mutant background, mutation of GAF/Trl binding sites in HOX-LacZ reporter genes) and had found no evidence for a role of GAF/Trl in Polycomb repression in larval tissues. It is fair to say that in most of the studies cited (i.e., references 12, 14, 49-52), the effect of GAF/Trl had been analyzed using PRE-miniwhite reporter gene activity as read-out, a much less stringent assay.

Thank you for pointing this out. Brown et al (reference 15) was overlooked when entering citations to these sections. We have added a sentence to the discussion to highlight the lack of necessity for Trl/GAF for imaginal disc silencing of Hox targets.

(5) The authors report that removal of GAF/Trl had almost no impact on H3K27me3 domain formation. Although this supports a model where PRC2 recruitment to PREs is unaltered after GAF/Trl depletion, it does not eliminate the potential caveat that PRC1 binding may be affected. Do the authors have binding profiles of PRC1 subunits or the H2Aub1 profile in embryos lacking GAF/Trl? It seems that the current paper would be a great opportunity to include such data and get them published. There is no need to generate PRC1 or H2Aub1 profiles if they don't already exist.

Unfortunately, we do not as of yet have any PRC1 reagents that are compatible with ChIP. Following the lack of effect of GAF on H3K27me3, we did not pursue the measurements of H2Aub in these knockdown conditions, given the likely dependence of H3K27me3 on H2Aub deposition at ZGA.

(6) Previous studies found that Polycomb group protein complexes bind to PREs in HOX genes both in cells where genes are OFF but also in cells where genes are ON but that the H3K27me3 profile is different in the two states, with H3K27me3 decorating the gene in the OFF but not in the ON state (Papp and Müller, Genes Dev 2006; Bowman et al, eLife 2014). In this study, the H3K27me3 profiles at target genes represent the sum of ChIP signals coming from cells where the gene is OFF and cells where the gene is ON. Is it known how eve and zen are deregulated in Zelda-depleted embryos? Could it be that the increased H3K27me3 enrichment at eve and zen is an indirect effect caused by loss of eve and zen expression in a large fraction of cells and consequently a gain of H3K27me3 ChIP signal at the gene in those cells? It may be worth at least discussing such scenarios in light of the studies mentioned above, and referring to them.

Thank you for raising this question. We have a manuscript in preparation that specifically addresses this issue, namely the relationship of on/off states to H3K27me3 deposition in the early embryo. In short, it is likely that the increase in H3K27me3 at *eve* reflects significantly reduced *eve* expression in *zelda* mutant embryos. We have added a clarifying sentence to the Discussion and added the indicated references.
